# Search for Effective Serum Tumor Markers for Early Diagnosis of Hepatocellular Carcinoma Associated with Hepatitis C

**DOI:** 10.17691/stm2021.13.1.03

**Published:** 2021-02-28

**Authors:** S.I. Malov, I.V. Malov, A.G. Kuvshinov, P.N. Marche, T. Decaens, Z. Macek-Jilkova, N.D. Yushchuk

**Affiliations:** Associate Professor, Department of Infectious Diseases, Irkutsk State Medical University, 1 Krasnogo Vosstaniya St., Irkutsk, 664003, Russia, Senior Researcher, Central Scientific Research Laboratory, Irkutsk State Medical Academy of Post-Graduate Education, a Branch of the Russian Medical Academy of Continuing Professional Education, 100 Yubileyny Microdistrict, Irkutsk, 664049, Russia; Professor, Head of the Department of Infectious Diseases, Irkutsk State Medical University, 1 Krasnogo Vosstaniya St., Irkutsk, 664003, Russia; Assistant, Department of Oncology and Radiation Therapy, Irkutsk State Medical University, 1 Krasnogo Vosstaniya St., Irkutsk, 664003, Russia; Professor, Vice Director of Research Center, Institute for Advanced Biosciences, Site Santé, Allée des Alpes, La Tronche, 38700, France; Professor, Research Director, Laboratory Head of Department of Hepatology and Gastroenterology, Centre Hospitalier Universitaire Grenoble Alpes, Avenue Maquis du Grésivaudan, La Tronche, 38700, France; Researcher, Department of Hepatology and Gastroenterology, Centre Hospitalier Universitaire Grenoble Alpes, Avenue Maquis du Grésivaudan, La Tronche, 38700, France; Professor, Academician of the Russian Academy of Sciences, Head of the Department of Infectious Diseases and Epidemiology, A.I. Yevdokimov Moscow State University of Medicine and Dentistry, 20/1 Delegatskaya St., Moscow, 127473, Russia

**Keywords:** hepatocellular carcinoma, hepatitis C, tumor markers, proteomics

## Abstract

**The aim of the study** was to identify the most effective serum tumor markers for early diagnosis of hepatocellular carcinoma based on the combination of diagnostic characteristics and correlations.

**Materials and Methods.:**

There were observed 55 patients with chronic hepatitis C in the stage of liver cirrhosis with a verified diagnosis of hepatocellular carcinoma. The control group consisted of 55 patients with chronic hepatitis C at the stage of liver cirrhosis without hepatocellular carcinoma, comparable to the experimental group in terms of basic clinical profile. The following tumor markers were estimated in both groups: alpha-fetoprotein (AFP), alpha-fetoprotein-L3 (AFP-L3), annexin A2 (ANXA2), heparin-binding growth factor Midkine (MDK), glypican-3 (GPC3), des-gamma-carboxyprothrombin (DCP, PIVKA-II), dickkopf-related protein 1 (DKK-1), osteopontin (OPN), and Golgi protein 73 (GP73). There were also evaluated such indices as diagnostic sensitivity, specificity, positive predictive value, negative predictive value, likelihood ratio of a positive test, the possible correlation between alpha-fetoprotein and other tumor markers. The area under the ROC curve (AUC) was calculated at the 95% confidence interval.

**Results.:**

The greatest sensitivity was revealed when using heparin-binding growth factor, annexin A2, osteopontin. Alpha-fetoprotein, alpha-fetoprotein-L3, glypican-3, des-gamma-carboxyprothrombin, dickkopf-related protein 1 had the best specificity. AUC>0.75 was found in annexin A2, heparin-binding growth factor, glypican-3, des-gamma-carboxyprothrombin, osteopontin, Golgi protein 73. The likelihood ratio of a positive test result was the highest for glypican-3. A significant correlation was found between alpha-fetoprotein and alpha-fetoprotein-L3, annexin A2, des-gamma-carboxyprothrombin.

**Conclusion.:**

According to the aggregate indicators of diagnostic efficiency, heparin-binding growth factor, glypican-3, and osteopontin are the most promising tumor markers of those studied. When they are used, integral AUC values are above the average, the level of these tumor markers in the blood of patients with hepatocellular cancer does not correlate with alpha-fetoprotein. They are applicable for diagnosing liver cancer in AFP-negative patients. The combined use of AFP + GPC3, AFP + OPN has already shown their advantages. However, the efficacy of the combination of AFP + MDK, GPC3 + OPN has not been determined yet; therefore, significance of the combined use of these tumor markers in the diagnosis of liver cancer should be investigated in the near future.

## Introduction

Hepatocellular carcinoma (HCC) ranks second among the causes of death in cancer patients worldwide [1]. The high rate is attributed to late diagnosis of the disease, as HCC is asymptomatic at an early stage and is detected only at the stage of tumor growth during ultrasound examination of the liver.

The main risk factor for the development of HCC is hepatitis B and C virus infection. Relevance of hepatitis B has decreased significantly due to introduction of the hepatitis B vaccine in the 90s and mass vaccination of the population. As a result, the focus in evaluation of etiological significance has shifted towards hepatitis C that has become the main infectious cause of HCC development today [2].

The first stage in the pathogenesis of liver cancer due to hepatitis C is supposed to be development of liver cirrhosis, which increases the risk of HCC manifold. For example, even after antiviral therapy and virus elimination, patients remain at risk of HCC, which is 2.1% per year in class A cirrhosis on the Child–Pugh score, and 7.8% per year in class B [3].

Therefore, improvement of methods for early detection of HCC is an urgent problem of healthcare today, solving it will provide the possibility to develop an effective system of patient care and reduce mortality from the disease.

In accordance with the clinical practice guidelines of the European Association for the Study of the Liver (EASL) [4] and clinical guidelines of the Russian Gastroenterological Association and the Russian Association of Oncologists [5], early diagnosis of HCC is based on ultrasound examination of the liver and measuring the level of serum alpha-fetoprotein (AFP) glycoprotein.

Abdominal ultrasound is widely used in medical settings, but its efficacy depends on the class of equipment, the doctor’s experience, and tumor size. The sensitivity of this method reaches 90% for tumors more than 5 cm in diameter, 70% for lesions with 1–2 cm diameter, and only 50% for those less than 1 cm in diameter [6].

The second diagnostic component is AFP synthesized by the endodermal cells of the embryonic yolk sac and subsequently by embryonic hepatocytes [7]. Increased AFP level in the blood serum is observed in various oncological diseases, but it is more significantly characteristic of HCC [8]. Analysis of the literature evaluating AFP as a biomarker of HCC showed that the range of its sensitivity and specificity equaled 26–65 and 80–94%, respectively, at different stages of HCC development [9, 10]. Due to low AFP sensitivity in some national versions of clinical guidelines, it is excluded from the diagnostic algorithm of HCC [11, 12].

Therefore, in recent years, all countries of the world have been actively searching for molecules and substances in the biological media of the body, detection of which would provide the possibility to make accurate diagnosis of HCC at an early stage. Research is carried out in the field of proteomics, genomics, and metabolomics. Detection of protein molecules is the most promising, since the methods for indicating various proteins are fairly well automated, highly sensitive, and reproducible.

**The aim of the study** was to identify the most effective serum tumor markers for early diagnosis of hepatocellular carcinoma based on the combination of diagnostic characteristics and correlations.

## Materials and Methods

We studied 110 patients with chronic hepatitis C (CHC) in the stage of liver cirrhosis, including 55 patients without signs of HCC and 55 with a verified diagnosis of HCC.

The diagnosis of hepatitis C was established on the basis of case history data, clinical examination, hepatic transaminase activity measurement, detection of anti-HCV IgG and hepatitis C virus RNA. The stage of liver fibrosis was determined using the FibroScan 502 apparatus (Echosens, France). Hepatitis C virus genotype 1 was detected in 56 patients (50.9%), genotype 2 — in 7 (6.4%), genotype 3 — in 47 (42.7%). These patients did not receive antiviral therapy in the past medical history. Liver cirrhosis was confirmed on the basis of clinical and laboratory data, liver elastometry, ultrasound, computed and/or magnetic resonance imaging. Severity of cirrhosis was determined using the Child–Pugh score [13, 14].

The diagnosis of HCC was established based on the EASL criteria [4]. The patients were kept under observation and treated at the Regional Clinical Infectious Disease Hospital, the Regional Clinical Consultative and Diagnostic Center, and the Regional Oncological Dispensary (Irkutsk, Russia). The diagnosis was verified morphologically in all patients. According to the TNM classification, stage I of the disease was detected in 10 individuals (18.2%), stages II–IIIA — in 45 (81.8%). This study was carried out in accordance with the Declaration of Helsinki (2013) and approved by the Ethics Committee of Irkutsk State Medical University (Russia). Written informed consent was obtained from each study participant.

Blood samples obtained before the surgical treatment or local tumor destruction procedure were used for laboratory studies. The group of patients with CHC was comparable to the group of patients with HCC in terms of the main clinical characteristics (Table 1).

**Table 1:** Clinical profile of patients with chronic hepatitis C with and without hepatocellular carcinoma (M±m)

Parameter	Patients with hepatocellular carcinoma (n=55)	Patients with chronic hepatitis C (n=55)	р
Average age (years)	59.9±4.5	57.7±10.5	>0.05
Gender, n (%):			
male	38 (69.1±6.2)	35 (63.6±5.7)	>0.05
female	17 (30.9±6.2)	20 (36.4±5.7)	>0.05
Abdominal pain, n (%)	10 (18.2±5.2)	8 (14.5±4.5)	>0.05
Weight loss, n (%)	45 (81.8±5.2)	45 (81.8±5.2)	>0.05
Average body mass index	25.7±11.0	23.8±8.0	>0.05
Fatigue, n (%)	52 (94.5±3.1)	45 (81.8±5.2)	0.038
History of blood transfusion, n (%)	7 (12.7±4.5)	6 (10.9±3.8)	>0.05
History of jaundice, n (%)	3 (5.4±3.1)	1 (1.8±2.0)	>0.05
Child–Pugh class, n (%):			
А	11 (20.0±5.4)	13 (23.6±5.7)	>0.05
В	25 (45.5±6.7)	22 (40.0±6.6)	>0.05
С	19 (34.5±6.4)	20 (36.4±6.5)	>0.05
Alcohol abuse (>16 points on the Audit score), n (%)	7 (12.7±4.5)	9 (16.4±4.5)	>0.05
Mean platelet count (×109/L)	124±40	138±50	>0.05
Total bilirubin, mean value (μmol/L)	47.9±18.7	27.2±8.6	>0.05
Albumin, mean value (g/L)	28.9±1.3	32.0±4.0	>0.05
ALT activity, mean value (IU/L)	76.6±33.8	65.5±10.9	>0.05
AST activity, mean value (IU/L)	98.5±40.1	88.0±9.8	>0.05
TNM, stage, n (%):			
I	10 (18.2±5.2)	-	-
II	37 (67.3±6.1)	-	-
IIIА	8 (14.5±4.6)	-	-

Follow-up care of patients with CHC was carried out for the average of 12 months and included clinical examination, general clinical and biochemical analyses, liver elastometry, abdominal ultrasound. If necessary, computed tomography or magnetic resonance imaging of the liver was performed. Thus, in the control group, the absence of HCC was confirmed for at least one year after blood sampling for tumor marker detection.

After blood collection, all serum samples were centrifuged and stored at –80°C.

The Architect *_i_*2000SR immunoassay analyzer equipped with chemiluminescence detection technology (Abbott Diagnostics, Korea) and Victor3 Plate Reader enzyme immunoassay analyzer (PerkinElmer, USA) were used to measure the level of tumor markers. The manufacturers of diagnostic kits and technical characteristics of test systems for tumor marker detection are presented in Table 2.

**Table 2:** Technical characteristics of test systems for detection of tumor markers used in the study

Tumor marker (its abbreviation)	Test system name; catalog number (manufacturer)	Sensitivity (ng/ml)
Alpha-fetoprotein (AFP)	Architect AFP; B3р360 (Abbott Diagnostics, Korea)	2.0
Alpha-fetoprotein-L3 (AFP-L3)	ELISA Kit for Alpha-Fetoprotein Lens Culinaris Agglutinin; SEB117Hu (Cloud-clone Corp., USA)	0.239
Annexin A2 (ANXA2)	ELISA Kit for Annexin A2; SEB944Hu (Cloud-Clone Corp., USA)	0.061
Heparin-binding growth factor Midkine (MDK)	ELISA Kit for Midkine; SEA63Hu (Cloud-Clone Corp., USA)	0.055
Glypican-3 (GPC3)	ELISA Kit for Glypican 3; SEA971Hu (Cloud-Clone Corp., USA)	0.057
Des-gamma-carboxyprothrombin (DCP, PIVKA-II)	Human protein induced vitamin K absence or antagonist-II (PIVKA-II) ELISA Kit; CSB-E13343h (Cusabio, China)	0.312
Dickkopf-related protein 1 (DKK-1)	ELISA Kit for Dickkopf-related protein 1; SEA74Hu (Cloud-Clone Corp., USA)	0.056
Osteopontin (OPN)	Human Osteopontin Platinum ELISA Kit; BMS 2066 (Affymetrix/ eBioscience, USA)	0.260
Golgi protein 73 (GP73)	ELISA Kit for Golgi protein 73; SEB668Hu (Cloud-Clone Corp., USA)	0.229

Laboratory studies were carried out at the Research Institute of Biomedical Technologies of Irkutsk State Medical University (Russia) and the Analytical Immunology Laboratory of the Institute for Advanced Biosciences of the Université Grenoble Alpes (France).

### Statistical processing methods.

Statistical processing was carried out using the Meta-DiSc 1.4 Software freely available (https://meta-disc.software.informer.com/1.4/). Statistical analysis included comparison of two samples and correlation analysis. The cut-off value for each tumor marker was found by calculating the highest Youden index value [15]. There were assessed such indicators as diagnostic sensitivity (Se), specificity (Sp), positive predictive value (PPV), negative predictive value (NPV), and positive likelihood ratio (PLR). Significance of differences between the studied indicators in the groups was determined using the chi-squared test (χ^2^) and Fisher’s exact test for four-field tables.

ROC analysis was used to assess the diagnostic efficiency of individual tumor markers [15]. The area under the ROC curve (AUC) was calculated at the 95% confidence interval (95% CI). AUC values were assessed according to the following criteria: AUC≤0.75 — low diagnostic efficiency; 0.75<AUC<0.90 — average; AUC≥0.90 — high. The differences were considered statistically significant at p≤0.05.

## Results

At the first stage, the optimal cut-off values were determined for each tumor marker according to the highest Youden index (Table 3). The selected cut-off value corresponded to the optimal balance of sensitivity and specificity. The highest sensitivity (≥80.0%) was revealed when using markers MDK, ANXA2, OPN for the diagnosis of HCC. At the same time, markers AFP, AFP-L3, GPC3, DCP (PIVKA-II), DKK-1, GP73 were characterized by the best specificity (≥80.0%) (Table 4).

**Table 3:** Optimal cut-off value and relationship between AFPs and other tumor markers

Tumor marker	Correlation coefficient (r)	Correlation coefficient (р)	Frequency of positive results at AFP<20 ng/ml (%)	Optimal cut-off (ng/ml)
AFP	—	—	—	20.0
AFP-L3	0.576	0.0003	13.3	13.5
ANXA2	0.337	0.048	33.3	16.0
MDK	0.241	0.16	73.3	0.8
GPC3	0.190	0.27	50.0	2.0
DCP, PIVKA-II	0.490	0.0029	20.0	20.0
DKK-1	0.272	0.11	43.3	1.2
OPN	0.145	0.41	66.7	100.0
GP73	0.262	0.13	53.3	1.2 IU/L

**Table 4:** Assessment of diagnostic value of hepatocellular carcinoma markers at the optimal cut-off

Tumor marker	Se, n (%)	Sp, n (%)	AUC	95% CI (р AUC)	PPV (%)	NPV (%)	PLR
AFP	25 (45.5)	52 (94.5)	0.630	0.57–0.70 (0.002)	89.3	63.4	8.27
AFP-L3	16 (29.1)	53 (96.4)	0.679	55.5–79.1 (0.002)	88.9	57.6	6.33
ANXA2	44 (80.0)	38 (69.1)	0.793	67.4–89.0 (0.001)	72.1	77.6	2.59
MDK	47 (85.5)	35 (63.6)	0.795	67.4–89.0 (0.001)	70.1	81.4	2.35
GPC3	33 (60.0)	53 (96.4)	0.836	72.1–92.5 (0.001)	91.7	70.7	16.67
DCP, PIVKA-II	30 (54.6)	49 (88.6)	0.760	64.4–86.6 (0.001)	83.3	66.2	4.79
DKK-1	28 (50.9)	44 (89.1)	0.707	58.4–81.7 (0.002)	71.8	62.0	4.67
OPN	44 (80.0)	42 (76.6)	0.787	74.1–83.5 (0.001)	77.2	79.3	3.42
GP73	35 (63.6)	44 (80.0)	0.764	64.4–86.6 (0.001)	76.1	68.8	3.18

Such integral index as AUC proved to be higher than the average in six markers out of nine under study: in ANXA2, MDK, GPC3, DCP (PIVKA-II), OPN, GP73. This characterizes them as potentially promising proteins in terms of diagnostic efficiency. At the same time, GPC3 had the highest PLR (see Table 4). This means that the probability of a positive test in CHC patients with HCC is 16.67 times higher than in CHC patients without HCC, which suggests significant diagnostic advantage of using this tumor marker.

To determine the effective combination of markers with AFP, it is important to know the degree of correlation between them. In the absence of correlation at AUC>0.75, each tumor marker makes additional contribution to the diagnostic efficiency without duplicating AFP indices. A significant correlation was found between AFP and AFP-L3, ANXA2, DCP (PIVKA-II), which throws the reliability of their combined use into question (see Table 3).

Another criterion for the selection of reliable diagnostic HCC tumor markers is the frequency of positive tumor marker detection in patients with AFP-negative HCC [10]. In this regard, MDK, OPN, GP73, and GPC3 showed the best results (see Table 3).

## Discussion

Recently, several dozens of tumor markers at various stages of clinical testing have been proposed for early diagnosis of HCC [16–18]. As follows from individual publications and comprehensive reviews, the use of specific proteins such as ANXA2, MDK, α-1-fucosidase (AFU), and immune complex squamous cell carcinoma antigen — IgM (SCCA-IgM) allows reaching 80% of the sensitivity level. However, diagnostic specificity of detecting these tumor markers is rather low at high sensitivity and varies from 50.0 to 70.5% [16, 19–22]. On the contrary, highly specific (>90%) protein markers (AFP-L3, plasminogen activator receptor (suPAR), DCP (PIVKA-II)) show low sensitivity of 28–76% [16, 22–25]. Thus, at present, no single tumor marker taken separately provides high diagnostic efficiency at the early stage of HCC [26].

For these reasons, the latest research has been directed towards the combined use of two, three, and even four tumor markers with different expression mechanisms in the process of carcinogenesis [27–30]. There have been described combinations including AFP, AFP-L3, DCP (PIVKA-II), ANXA2, OPN, DKK-1, receptor tyrosine kinase sAxl (AXL), thioredoxin (Trx1) [22, 26, 27, 31, 32]. In most cases, this provides the possibility to improve diagnostic efficiency in some way. However, combining certain tumor markers is usually carried out intuitively, in an arbitrary manner. Besides, it should be taken into consideration that, methodologically, many studies were performed using heterogeneous clinical groups in terms of HCC etiology. Research often includes patients at different stages of the disease. Obviously, the presence of patients who are both at an early and advanced disease stages in groups under study does not allow assessing the value of markers specific for early diagnosis of HCC (stages I–II according to the TNM classification). Moreover, when tumor size is more than 2.0 cm, instrumental diagnostic methods (ultrasound, computed, and magnetic resonance imaging) are quite effective, and there is no need to detect serum tumor markers [4].

The present study has investigated the most informative parameters influencing the diagnostic efficiency of tumor markers and the possibility of their combined use with AFP (Table 5).

**Table 5:** Indices of diagnostic efficiency of tumor markers

Tumor marker	Index				Number of indices of diagnostic advantages
	Absence of significant correlation with AFP	AUC>0.75	PLR>4.0	More than 60% of positive results in patients with AFP<20 ng/ml	
AFP	—	—	+	—	—
AFP-L3	—	—	+	—	1
ANXA2	—	+	—	—	1
MDK	+	+	—	+	3
GPC3	+	+	+	—	3
DCP, PIVKA-II	—	+	+	—	2
DKK-1	+	—	+	—	2
OPN	+	+	—	+	3
GP73	+	+	—	—	2

MDK, GPC3, and OPN proved to have the greatest diagnostic advantages. Separately, these tumor markers have already been tested to various extents in the diagnosis of HCC [17, 19, 26, 33].

MDK is a growth factor stimulating cell proliferation and differentiation. It was established that MDK levels in HCC patients were on average 5 times higher than in those with liver cirrhosis without HCC. The Se for isolated MDK was 90% and for AFP only 50% [19].

GPC3 belongs to the glypican-proteoglycan family. An elevated GPC3 level is revealed in 50–55% of HCC patients and only in 5% of those with liver cirrhosis [34]. The independent significance of GPC3 is limited for the diagnosis of HCC due to its low sensitivity [35]. GPC3 is detected immunohistochemically in liver biopsies, which is used clinically in the differential diagnosis of HCC and other liver lesions [36].

OPN is an integrin-binding glyco-phosphoprotein produced in increased amounts in many malignant neoplasms [37]. The OPN level elevates 6–12 months before the instrumental detection of HCC and has better sensitivity than AFP [38].

The level of these tumor markers in the blood of HCC patients does not correlate with AFP. When they are used, integral AUC values are above the average, they are applicable for diagnosing HCC in AFP-negative patients. Moreover, GPC3 differs from other tumor markers by a significantly higher PLR index, which makes it promising for combined use.

The combined use of AFP + GPC3 and AFP + OPN has already demonstrated their advantages (AUC is 0.85 and 0.90, respectively) [29, 33]. However, MDK and combinations of AFP + MDK, GPC3 + OPN have not been studied yet, therefore, it is a question of future research to investigate effectiveness of using these tumor markers in combination.

## Conclusion

The study has revealed candidate proteins, quantitation of which in the blood serum of patients with chronic hepatitis C has a number of diagnostic advantages over other tumor markers. MDK, GPC3, and OPN are the most promising among them. ROC analysis and study of correlations give reasons to expect highly effective results from using tumor markers in combination with AFP and with each other.
